# PhoXplex: Combining Phospho-enrichable Cross-Linking with Isobaric Labeling for Quantitative Proteome-Wide Mapping of Protein Interfaces

**DOI:** 10.1021/acs.jproteome.4c00567

**Published:** 2024-10-18

**Authors:** Runa D. Hoenger Ramazanova, Theodoros I. Roumeliotis, James C. Wright, Jyoti S. Choudhary

**Affiliations:** † Functional Proteomics team, Chester Beatty Laboratories, The Institute of Cancer Research, London SW3 6JB, United Kingdom

**Keywords:** cross-linking mass spectrometry (XL-MS), enrichable cross-linker, PhoX, TMT, cancer cell lines, mutations, protein interactions

## Abstract

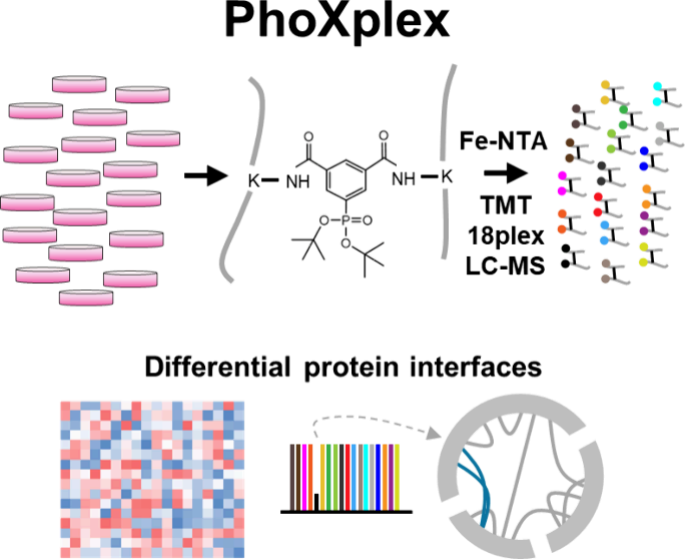

Integrating cross-linking mass spectrometry (XL-MS) into structural biology workflows provides valuable information about the spatial arrangement of amino acid stretches, which can guide elucidation of protein assembly architecture. Additionally, the combination of XL-MS with peptide quantitation techniques is a powerful approach to delineate protein interface dynamics across diverse conditions. While XL-MS is increasingly effective with isolated proteins or small complexes, its application to whole-cell samples poses technical challenges related to analysis depth and throughput. The use of enrichable cross-linkers has greatly improved the detectability of protein interfaces in a proteome-wide scale, facilitating global protein–protein interaction mapping. Therefore, bringing together enrichable cross-linking and multiplexed peptide quantification is an appealing approach to enable comparative characterization of structural attributes of proteins and protein interactions. Here, we combined phospho-enrichable cross-linking with TMT labeling to develop a streamline workflow (PhoXplex) for the detection of differential structural features across a panel of cell lines in a global scale. We achieved deep coverage with quantification of over 9000 cross-links and long loop-links in total including potentially novel interactions. Overlaying AlphaFold predictions and disorder protein annotations enables exploration of the quantitative cross-linking data set, to reveal possible associations between mutations and protein structures. Lastly, we discuss current shortcomings and perspectives for deep whole-cell profiling of protein interfaces at large-scale.

## Introduction

Cross-linking mass spectrometry (XL-MS) methods utilize bifunctional chemical reagents that covalently bridge proximal protein regions to preserve the structural attributes of proteins and protein assemblies in their native state.^1−6^ Following proteolytic cleavage, the cross-linked residues are identified by mass spectrometry and eventually mapped on structural models in the three-dimensional space using bioinformatics analysis. As the cross-linker molecule has a finite length, restraints in the Euclidean distances between cross-linked residues can be imposed to assist the evaluation of proposed structural models, such as those computationally predicted by AlphaFold.^7−10^ Due to its versatility and ease of implementation, XL-MS has been widely used to validate or refine protein structures and to inform topological mapping of protein assemblies in isolated proteins and samples with low complexity in a qualitative fashion.^11−13^ Specifically, the integration of XL-MS data into deep learning methods has been shown to significantly improve modeling performance at the level of single proteins and multisubunit complexes.^14,15^

Advancements in cross-linker chemistries, sample preparation, mass spectrometry, and data analysis approaches have more recently expanded the applications of XL-MS to complex biological samples to enable the characterization of protein–protein interactions at the interface resolution in a proteome-wide scale. These include whole cells,^1,16−19^ subcellular components,^20−24^ and tissues.^25^ Data from many of the whole proteome cross-linking studies from various sources, cell models, chemistries, and analytical and statistical approaches have been consolidated into a repository to enable the holistic analysis of XL-MS derived protein interactions.^26^ However, the system-wide application of XL-MS remains challenging with a very low percentage of cross-linked peptides reported in the whole proteome digest that is mainly dominated by regular peptides. To enhance the detection of cross-linked peptides in complex samples, several cross-linkers that accommodate enrichable handles in their structures have been developed in MS-cleavable or noncleavable formats.^19,27−31^ Among these, the NHS ester-based PhoX cross-linker that contains a phosphonic acid group makes the cross-linked peptides amenable to immobilized metal affinity chromatography (IMAC) enrichment.^29,32^ While this non-MS-cleavable cross-linker does not benefit from the advantage of MS-cleavable reagents in data processing in large-scale studies, the strategy deployed by PhoX is a particularly attractive one due to the simplicity of the enrichment that is based on well-established and robust methods involving a small number of steps.

The combination of XL-MS with peptide quantitation techniques utilizing differential labeling with isotopically coded reagents is a powerful technique to elucidate the dynamics of protein interfaces across different cellular states.^33−40^ This strategy can discern sample specific conformational alterations and disruption of protein interfaces as well as map interactomes in a massive parallel way. When combined with high-capacity isobaric labeling proteome analysis, this comparative approach can reveal another distinct layer of protein attributes beyond protein abundances and post-translational modifications at large-scale. Several studies, including previous work from our laboratory, have explored the basal proteome characteristics of large panels of cancer cell lines and have derived protein networks by coregulation analysis,^41,42^ however these lack structural information about protein interfaces. Moreover, the ability to detect potential structural variations is also pivotal in understanding the consequences of genomic alterations on protein functions across variable genotypes.

In this study we combined phospho-enrichable cross-linking with TMT-based peptide quantification to develop a streamline workflow (PhoXplex) that can facilitate the detection of differential structural features across different cell lines or conditions in a proteome-wide scale. Application of the workflow in a panel of colorectal cancer cell lines yielded the relative quantification of over 9000 cross-links and long loop-links in total, making this the deepest TMT-based quantitative XL-MS study in whole cells to date. We investigated the agreement of the identified cross-links with AlphaFold models as well as their overlap with disordered protein regions and leveraged cross-linking quantitation to showcase possible associations between genomic mutations and protein structures. Lastly, we discuss current limitations and future directions for deep large-scale comparative profiling of protein interfaces.

## Methods

### Experimental Design Overview

**Figure 1:**
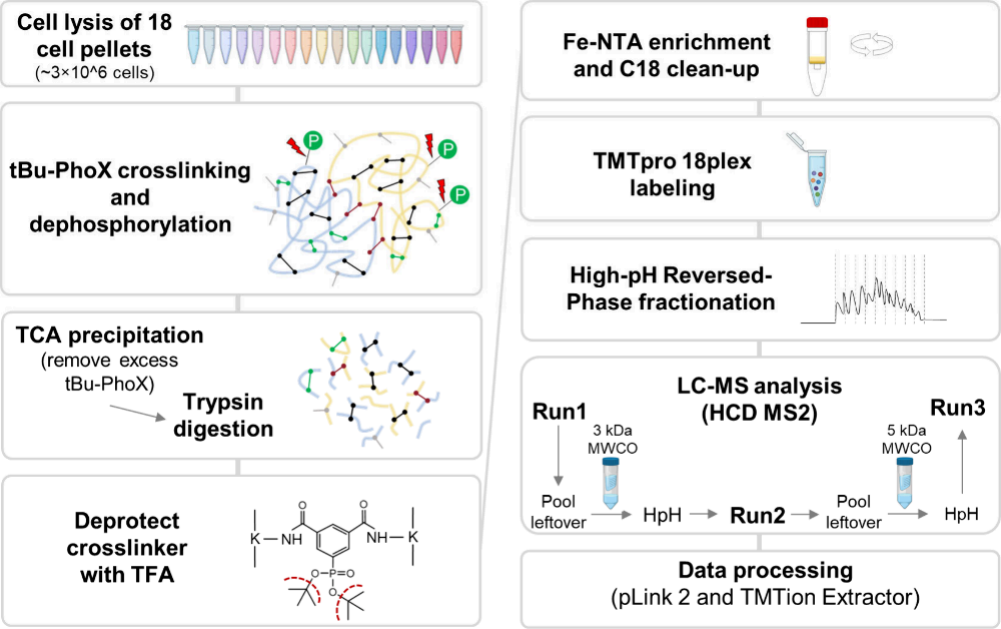
Schematic overview of the PhoXplex workflow for quantitative proteome-wide mapping of protein interfaces.

Cell pellets were lysed in a nondenaturing buffer supplemented with a nuclease using brief probe sonication on ice. Equal amounts of total protein in the crude lysates were cross-linked with the *tert*-butyl disuccinimidyl phenyl phosphonate cross-linker (TBDSPP, tBu-PhoX). Although in this feasibility study we employed cell lysates in a nondenaturing buffer, we utilized a cell permeable cross-linker to ensure that the method remains adaptable for use in live cells with only minimal adjustments to the current protocol. Additionally, the enhanced permeability aids in cross-linking unsolubilized cellular components in the lysates remaining after brief sonication. Using lysates also permits parallel cross-linking reactions across multiple cell lines, a crucial factor for maintaining reproducibility. This approach is particularly advantageous in large-scale studies involving diverse cell lines, where differences in growth rates could complicate the synchronization of cross-linking reactions. After quenching the cross-linking reaction, the samples were incubated with a phosphatase overnight to remove all endogenous protein phosphorylation. Samples were further lysed by stronger probe sonication, and proteins were precipitated with TCA to remove the excess of the cross-linker. Protein pellets were digested with trypsin, and the digests were acidified to remove the *tert*-butyl groups from the cross-linker and expose the enrichable phosphate group. Samples were subjected to Fe-NTA enrichment, and the eluates were labeled with the TMTpro 18plex reagents. Peptide cleanup with C18 spin columns was performed before and after the enrichment. The enriched peptides were fractionated offline with high-pH reversed phase chromatography and analyzed with an MS2 method using HCD fragmentation on an Orbitrap Ascend mass spectrometer. To maximize the MS sampling of low abundant cross-linked peptides, we did two additional series of injections of the leftover peptides from the first run (run1) after pooling, ultrafiltration with 3 kDa (run2) and sequentially with 5 kDa (run3) molecular weight cutoffs, and refractionation of the retained peptides. Raw data were processed in pLink2^43^ software for the identification of cross-linked peptides. TMTpro quantification was performed with an in-house-made Python script (TMTionExtractor), which retrieved TMTpro peak intensities from the pLink2 generated mgf files by matching the spectrum IDs in the results reports. An overview of the main steps of the PhoXplex workflow is shown in Figure 1.

### Cell Lines

For the colorectal cancer cell lines, frozen cell pellets collected during a previous study from our group were used and are described in Roumeliotis et al.^41^ Briefly, cells were grown in either a DMEM/F12 medium (Gibco) supplemented with 10% fetal calf serum (v/v) (Gibco) and 50 U/mL penicillin and 50 mg/mL streptavidin (Gibco), or a RPMI 1640 medium (Gibco) supplemented with 10% fetal calf serum (v/v) (Gibco), 50 U/mL penicillin, 50 mg/mL streptavidin (Gibco), 2.5 mg/mL glucose (Sigma), and 1 mM sodium pyruvate (Gibco), and maintained at 37 °C in a humidified atmosphere at 5% CO_2_. The cells were harvested by incubation with TrypLE (Gibco) until detached and washed twice with a cold DPBS solution before snap freezing on dry ice. Each pellet contained approximately 3 × 10^6^ cells.

### TBDSPP (tBu-PhoX) Cross-Linking

Cell pellets were lysed in 400 μL of a 20 mM HEPES, 150 mM NaCl, pH 7.5 buffer supplemented with 4 μL of Halt Protease Inhibitor Cocktail (Thermo, #78429) and 0.5 μL of universal nuclease (Pierce, #88701) with 10 s probe sonication at a pulse of 1 s every 3 s at amplitude 30% (EpiShear) on ice. Bradford assay was performed to normalize the protein amount to 0.7 mg for every cell line and each sample was diluted up to 490 μL with the lysis buffer (∼1.4 μg/μL). A 50 mM cross-linker stock solution was prepared by dissolving 5.5 mg of TBDSPP (Thermo, A52287) in 200 μL of extra dry acetonitrile. A TBDSPP stock solution was added in each cell lysate slowly and mixed quickly with a pipet tip while adding, at a final concentration of 1 mM (10 μL). Samples were incubated at room temperature for 1 h, and the reaction was terminated by adding 1 M ammonium bicarbonate to a final concentration of 20 mM to each sample, followed by incubation for 15 min before adding 5 μL of alkaline phosphatase (Pierce, #31392), and incubating in a thermomixer at 37 °C at 500 rpm overnight for protein dephosphorylation.

### Trypsin Digestion

To fully lyse the cells after the cross-linking, the samples were probe sonicated again at 35% amplitude for 15 s with a pulse every 1 s. Cysteines were reduced with tris-2-carboxyethyl phosphine (TCEP) at a final concentration of 5 mM and alkylated with freshly prepared iodoacetamide at a final concentration of 10 mM, simultaneously at room temperature for 1 h. To remove the excess of the cross-linker, proteins were precipitated with the addition of 100 μL of 10 M trichloroacetic acid (TCA), vortexed, and followed by centrifugation at 4 °C for 10 min at 20,238 × g. Supernatants were discarded, and 300 μL of 100% cold acetone was added to the protein pellets, which were bath sonicated for 1 min, followed by another centrifugation as above. The supernatant was discarded, and 200 μL of a 100 mM triethylammonium bicarbonate (TEAB) buffer was added. For digestion of the protein pellets, a trypsin solution of 500 ng/μL (Pierce #90058) was added in each sample at a final concentration of 50 ng/μL and samples were incubated at 37 °C with shaking at 900 rpm for 16 h. To deprotect the cross-linker prior to phospho-enrichment, 6.6 μL of 100% TFA was added (3%) and incubated for 1 h at 37 °C in a thermomixer set at 500 rpm. All samples were then dried in a SpeedVac concentrator, and peptides were cleaned-up with desalting spin columns (Pierce, #89851) and dried again before the phospho-enrichment.

### Phosphoenrichment and TMTpro Labeling

Phospho-enrichment was performed using the High-select Fe-NTA phosphopeptide enrichment kit according to manufacturer’s protocol (Thermo, A32992). Briefly, after resuspending the peptides in 200 μL of Binding/Wash buffer, and equilibrating the spin columns, peptide mixtures were incubated in the spin columns for 30 min with gentle mixing of the resin with the sample every 10 min. After binding, the columns were centrifuged for 30 s at 1000 × g to collect the flow-through into clean Eppendorf tubes. The resin was then washed to remove nonspecific adsorbed peptides. The bound phospho-containing peptides were eluted using the elution buffer by centrifugation at 1000 × g for 30 s and the eluates were SpeedVac dried. After phospho-enrichment, peptide C18 cleanup (Pierce, #89851) was performed to remove any residual ammonia from the elution buffer, and samples were SpeedVac dried. Samples were reconstituted in 25 μL of 100 mM TEAB and labeled with 10 μL of TMTpro-18plex reagents dissolved in extra dry acetonitrile (TMTpro: 25 μg/μL, Thermo). Reaction was terminated with 2 μL of 5% hydroxylamine for 15 min at room temperature. Labeling efficiency was verified with a premix LC-MS run (pool of 0.5 μL from each sample) prior to further processing.

### High-pH Reversed-Phase Peptide Fractionation

Offline peptide fractionation was based on high-pH reversed-phase (RP) chromatography using the Waters XBridge C18 column (2.1 × 150 mm, 3.5 μm) on a Dionex UltiMate 3000 HPLC system at a flow rate of 0.2 mL/min. Mobile phase A was 0.1% (v/v) ammonium hydroxide, and mobile phase B was acetonitrile and 0.1% (v/v) ammonium hydroxide. Pooled TMTpro-peptides were resuspended in 100 μL of buffer A, centrifuged at 18,407 × g for 5 min, and the supernatant was injected for fractionation with the following gradient: isocratic for 5 min at 5% phase B, up to 15% B in 3 min, gradient for 32 min to 35% phase B, gradient to 90% phase B in 5 min, isocratic for 5 min, and re-equilibrated to 5% phase B. Fractions were collected every 30 s, SpeedVac dried, and pooled into 40–50 samples for MS analysis for each fractionation.

### Ultrafiltration of Peptides

One third of the high-pH fractions was analyzed with LC-MS for identification of cross-linked peptides and quantification of proteins using the linear and monolinked peptides from the same raw files. The remaining peptide solutions were pooled again in an Eppendorf tube and subjected to ultrafiltration followed by high-pH reversed phase fractionation of the high MW part to enhance the MS sampling of cross-linked peptides in the repeated LC-MS runs. Specifically, the pooled sample was dissolved in 1 mL of 10% acetonitrile, transferred into the Amicon 3000 MWCO cartridge (Sigma, UFC9003) and centrifuged at room temperature for 50 min at 4000 × g. The buffer was exchanged two more times with 1 mL of 10% acetonitrile and centrifuged at room temperature for 30 min at 5000 × g each time. The top and bottom fractions were transferred to different Eppendorf tubes and SpeedVac dried before a second fractionation and further LC-MS analysis. The process was repeated also using 5000 MWCO (VIVASPIN 500); the sample was diluted with 200 mL of 5% acetonitrile, transferred to the cartridge, and centrifuged at room temperature. The buffer was exchanged two more times.

### LC-MS Analysis

The analysis of peptides was performed by online nano-LC-MS/MS on an Orbitrap Ascend Tribrid mass spectrometer coupled with an UltiMate 3000 RSLCnano System (Thermo). Samples were first loaded on a trap column (100 μm i.d. × 2 cm, PepMap C18, 5 μm, 100 Å) at 10 μL/min with 0.1% TFA loading buffer for 3 min and then separated on an analytical column (Waters, nanoE MZ PST BEH130 C18, 1.7 μm, 75 μm × 250 mm) over an 80 min linear gradient of 3–29% of 80% CH3CN/0.1% formic acid at 300 nL/min. The analytical column was connected to an EASY-Spray emitter (Thermo, ES991) on an EASY-Spray source. The Orbitrap Ascend was operated in a data-dependent acquisition top speed mode (3 s) targeting peptide precursors with charge states +3 to +8. MS scans were acquired with a resolution of 120,000 within an *m*/*z* range of 380–1400. MS2 spectra were acquired with HCD fragmentation (higher collision dissociation) with collision energy 36% and a quadrupole isolation width of 0.7 Th (Thomson unit), detection in the Orbitrap with 45000 resolution, 200% normalized AGC, and maximum injection time of 91 ms. Targeted precursors were dynamically excluded from further isolation and activation for 45 s with 10 ppm mass tolerance.

### Data Processing

Raw LC-MS files were processed with pLink version 2.3.11 for identification of cross-linked, loop-linked, monolinked, and regular peptides. Precursor and fragment mass tolerances were 20 ppm and 0.02 Da, respectively. The linker mass of PhoX (+209.972 Da) with a mono mass of +227.982 Da (PhoX hydrolyzed) targeting Lysine and protein N terminus were configured and used for the search. Additionally, Carbamidomethyl at C (+57.021464 Da) and TMTpro (+304.207146 Da) at the peptide N terminus were selected as fixed modifications, as well as TMTpro (+304.207146 Da) at K as variable modification. Spectra were searched against a fasta file containing 9405 UniProt entries comprising the COREAD cell line proteome as previously determined,^41^ for tryptic peptides with up to 3 missed-cleavages and minimum peptide length of 5. Cross-links were filtered at PSM level FDR < 1% separately for intra- and interlinks with 20 ppm tolerance. To identify high confidence protein–protein interactions (PPIs), the pLink search was repeated with the same settings but using a fasta file containing the sequences of all proteins for which we found cross-links and long loop-links in the first search (true target) concatenated with a shuffled decoy version of these (false target). The xiFDR^17^ software was used to filter for PPIs at FDR < 10% from the CSMs output of the second search. Cross-linked peptides containing single peptides mapping to multiple proteins were excluded from the xiFDR input file. The raw files were also processed with Proteome Discoverer v3.0 (Thermo) using the Sequest HT search engine for identification and TMTpro-based quantification of linear peptides and monolinks to obtain protein level relative quantification across the cell lines to correct the cross-linking quantification for baseline protein expression. The settings included: MS1 and MS2 ion mass tolerances of 20 ppm and 0.02 Da respectively, maximum number of missed-cleavages 2; Carbamidomethylation of cysteines and TMTpro at peptide N terminus as static modifications. Dynamic modifications were Oxidation of M (+15.995 Da), Deamidation of N/Q (+0.984 Da), TMTpro at K (+304.207 Da), Phosphorylation of S/T/Y (+79.966 Da), PhoX Hydrolysed at K (+227.982 Da) and PhoX Amidated at K (+226.998 Da). Data were searched against UniProt reviewed *Homo sapiens* protein entries. Peptide FDR was set at 0.01 and validation was based on q-value and target-decoy database search. Only unique peptides were used for quantification, considering protein groups for peptide uniqueness. Peptides with an average reporter signal-to-noise greater than 3 were used for protein quantification.

### TMTionExtractor Tool Description

TMTionExtractor (https://github.com/DrJCWright/TMTion_Extractor) is a python-based tool for extracting TMT ion intensities from MGF formatted MS2 spectra and additionally mapping them to pLink2^43^ output. The user inputs a directory of MGF files to process and can optionally set parameters for TMT ion matching tolerance in ppm, the output file name, and pLink2 csv output files, including cross-linked peptide results, into which TMT ion intensities can be mapped. There is a *beta* option to normalize TMT intensities based on total summed intensity for each TMT channel and then scale the normalized intensities across samples. This approach is not recommended for fractionated data or intentionally unbalanced sample mixing, as it assumes that the total amount of TMT signal is equal and the total abundance of peptides from each sample to be equal.

In brief, this tool parses each MGF in the user defined directory and matches peaks in the low mass region to a dictionary of known TMT ion *m*/*z* values. TMT peaks are matched within a default 15 ppm tolerance. Tolerances greater than 20 ppm are not recommended as this is over half the *m*/*z* difference between TMT ions and could lead to overlapping peaks being integrated together. In rare cases, multiple peaks are matched within tolerance; these are summed together. A tab delimited table is written to the file, with each row representing an MS2 spectrum and the following columns: mgf file name, spectrum title, raw TMT ion intensities, absolute delta *m*/*z* for the between matched peak and reference monoisotopic mass of the TMT label. Additional columns may include normalized and scaled abundance values for the TMT ion intensities.

If compatible pLink2 csv files have been specified, these will be parsed by the tool. Protein and peptide headers will be extracted while PSM rows are parsed. Using the TMT ion table, PSMs are matched to spectra and TMT intensities are appended. TMT ion intensities for multiple PSMs matching a peptide or protein are summed and appended to the protein/peptide header. A new pLink2.csv file is written with the mapped and appended TMT ion intensities.

### Data Analysis and Visualization

Raw TMTpro intensities of cross-linked and loop-linked peptide spectra (with a distance of greater than or equal to 5 amino acids) were aggregated at the unique residue pair level by summing the intensities. For each sample, the total sum of these intensities was computed. Normalization factors were then derived by dividing each sample’s sum by the maximum sum value across all samples. Finally, the cross-link intensities were normalized by dividing them by the corresponding normalization factors. The data were then normalized by scaling each row to its mean value, followed by a log2 transformation (resulting in scaled data). For the total proteome quantification, the raw abundances were exported from Proteome Discoverer and normalized and scaled in the same way as the cross-linking data. To regress-out the protein profiles from the respective cross-linking profiles of intralinks only, protein values were used as the independent variables (x) and the cross-linking values were used as the dependent variables (y) of the linear models; the residuals of the models were the net cross-linking differences. Bar plots and pie charts were done in Excel, Venn diagrams were plotted in the BioVenn application^44^ and adapted in PowerPoint, histograms and boxplots were made in RStudio, heatmaps were done with the Phantasus^45^ and TBtools^46^ applications. The protein network was visualized in Cytoscape 3.9.1.^47^ Cross-link maps on 2D sequences, 3D structures and distances were visualized and computed in the xiView platform.^48^ Protein structure models were downloaded from the AlphaFold portal (v4). Mutation data for the colorectal cancer cell lines were downloaded from the COSMIC database.^49^ Only missense mutations were used and compiled as a binary table where values of 1 represented at least one mutation in each cell line and values of 0 showed the absence of missense mutations. These were consolidated with the regressed cross-linking data, and the mutation positions were compared with position1 and position2 of the cross-links to identify overlaps using an R script and the TEXTJOIN function in Excel that was first used to aggregate mutation positions per gene (e.g., MCM7 | LS411N_284; SNU1040_611; SNU1040_353; HCC2998_231). Curated disordered protein regions were downloaded from the DisProt repository^50^ and cross-links within the start and end positions of the disordered regions were mapped with an R script. Protein classification analysis was done with the PANTHER knowledgebase.^51^ Per-residue model confidence scores (pLDDT) were computed with an R script using the bio3d package^52^ and the AlphaFold_v4 models.

## Results

**Figure 2:**
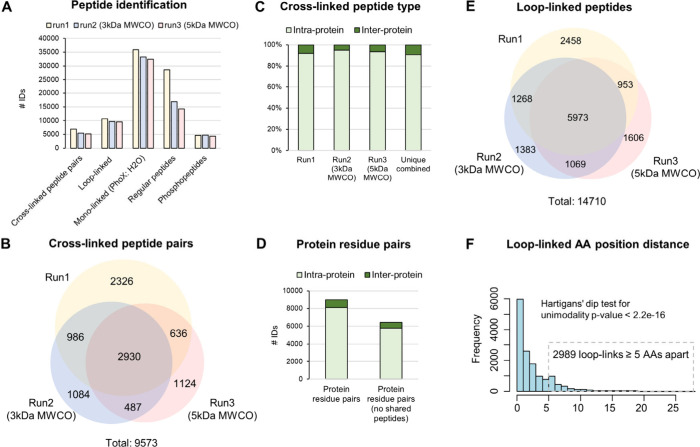
Summary of the peptide data generated. A) Bar plot showing the number of cross-linked, loop-linked, monolinked, regular, and phosphorylated peptides identified in each analysis. B) Venn diagram illustrating the overlap of cross-linked peptides between the three runs based on MW ultrafiltration. C) Stacked bar chart showing the percentages of intra- and interlinked peptides. D) Stacked bar chart of intra- and interlinked protein residue pairs. E) Venn diagram showing the overlap of loop-linked peptides between the three runs based on MW ultrafiltration. F) Histogram of the position distance on the protein sequence of loop-linked peptides expressed as the number of amino acids between the linked positions.

To develop a streamlined workflow for quantitative proteome-wide mapping of protein interfaces, we combined phospho-enrichable protein cross-linking with peptide isobaric labeling by TMT-18plex (PhoXplex). For this feasibility study we used frozen cell pellets of ∼3 million cells each from 14 colorectal cancer cell lines (COREAD) which we previously characterized at the basal proteome level.^41^ For the four cell lines, we used duplicate cell pellets to assess the reproducibility of the workflow. Application of the PhoXplex workflow resulted in the identification of several types of peptides that are summarized in Figure 2A. In each round of MS analysis before and after MW cutoff filtering, we identified over 5000 cross-linked and 9500 loop-linked peptides (Supporting Information, Table S1 and Table S2), as well as over 30,000 monolinked peptides (hydrolyzed PhoX dead-ends), ∼14,000–28,000 regular peptides and ∼4500 phosphopeptides. Of note, the number of detected phosphopeptide was relatively small considering the scale and fractionation depth of the experiment, suggesting high efficiency of the added phosphatase. As expected, the number of the identified regular peptides decreased with an increased ultrafiltration MW cutoff (Figure 2A) as only larger peptides were retained more efficiently (Figure S1A). Surprisingly this was not the case for monolinked peptides, possibly because they were slightly bigger than regular tryptic peptides due to higher missed-cleavage rates on modified lysines (Figure S1A). This also suggests that the ultrafiltration devices retain peptides with molecular weights smaller than expected, possibly due to other physiochemical properties. Nevertheless, the additional MW-based separations helped to enhance the sampling of targeted precursor ions leading in the total identification of 9573 unique cross-linked peptide pairs (Figure 2B). Of these, 90.8% and 9.2% represented intra- and interprotein links, respectively (Figure 2C). Notably, the proportion of interlinks is significantly lower than that previously found using the DSSO cross-linker in intact subcellular organelles (∼25%).^20^ This is likely due to the shorter length of the PhoX cross-linker (4.8 Å) and/or its noncleavable nature with consequent differences in FDR estimation.^53^ Mapping of the cross-linked peptides to protein sequence positions resulted in 8987 protein residue pairs. Further removal of cross-links containing at least one peptide that is shared between proteins resulted in 6447 unique protein residue pairs (Figure 2D).

Additionally, we identified 14710 unique loop-linked peptides from all three runs combined (Figure 2E). These represent cross-linked proximal lysines within the same peptide sequence. To evaluate the distance characteristics of the loop-linked lysines on the primary protein structure, we plotted the distribution of their position differences expressed as the number of amino acids between the linked positions. This revealed a bimodal distribution with a second peak at five amino acids or longer (Figure 2F), encompassing 2989 intrapeptide links. These likely have distinct and structurally informative characteristics as opposed to those in neighboring positions. Taken together, we compiled a list of 9151 nonredundant protein residue pairs from cross-linked and long loop-linked (≥5 AA distance) peptide types mapping on 2797 proteins. Protein classification analysis showed that the cross-linked proteins mainly represent metabolic, translational, cytoskeletal, scaffold, transport, transcriptional and chromatin-binding related proteins (Figure S1B).

Although we used separate FDR for intra- and interlinks, heteromeric cross-links are more prone to random matching due to their relative rarity and the larger search space created by the increased number of possible theoretical combinations.^54,55^ To identify a set of high confidence PPIs, we performed a separate search of all raw data in pLink2 using a fasta file containing the sequences of all proteins for which we found cross-links and long loop-links in the first search (true target) concatenated with a shuffled decoy version of these (false target) and utilized the xiFDR^17^ software to filter for PPIs at <10% FDR. We followed this entrapment database approach as pLink2 does not report the internally generated reversed decoy hits in the output and therefore is not directly compatible with xiFDR. Although this approach can enhance the reliability of the reported PPIs, it can potentially inflate the FDR by increasing the search space and the likelihood of random matches. We identified 216 protein–protein interactions (PPIs) in a subset of 302 proteins (Figure S1C and Table S3). Notably, only 53% of the PPIs are catalogued in STRING^56^ as physical interactions, revealing a significant number of potentially novel physical associations that occur across multiple cell types.

**Figure 3:**
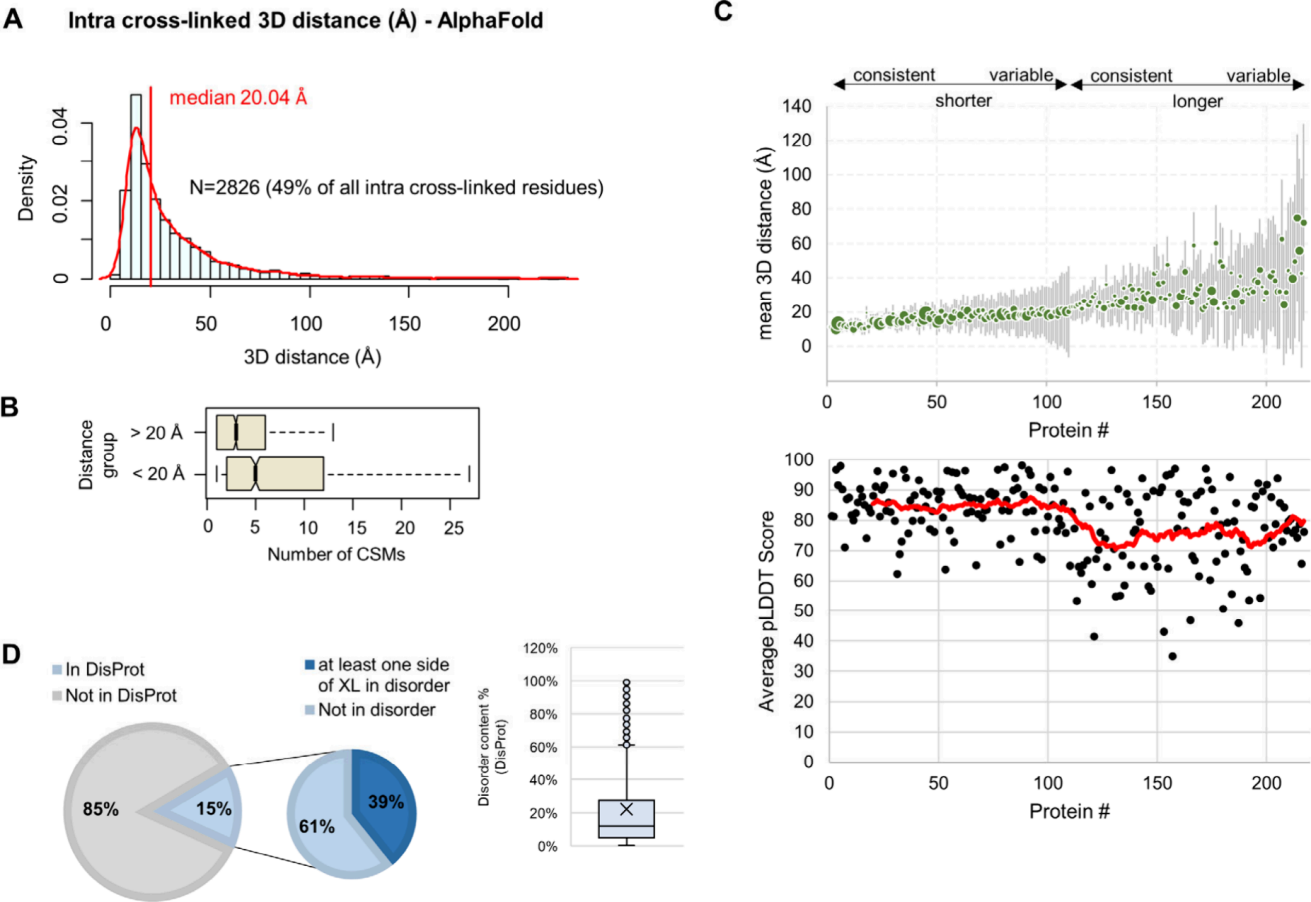
Mapping of cross-links on AlphaFold models and disordered protein regions. A) Histogram of Euclidean distances of 2826 intralinks from 217 proteins with at least 6 intralinks mapped on AlphaFold structures. B) Boxplots of cross-link spectrum matches (CSMs) for cross-links with 3D distances smaller or greater than 20 Å. C) Top panel: Dots show the mean 3D distance for each one of the 217 proteins with at least 6 intralinks. The proteins are classified into two groups, “shorter” and “longer” for mean distance <20 Å and >20 Å, respectively. Proteins with “shorter” mean distance are in better overall agreement with the AF models compared to those with “longer” average distances. The variation within each protein’s intralink distances is expressed as standard deviation (SD) and is shown as error bars. The dots are sorted according to SD from smallest to largest, within each one of the “shorter” and “longer” groups. Additionally, the size of dots is proportional to mean number of CSMs per protein. Bottom panel: Dot plot of average per-residue model confidence scores (pLDDT) for AlphaFold predictions for each of the 217 proteins. Red line shows the moving average of data points for every 20 points. This shows that the “longer” group has a trend for lower confidence predictions. D) Pie charts showing the percentage of cross-links for which at least one protein in the pair is found in the DisProt database. In this subset, a second pie chart shows the percentage of cross-links with at least one side linked to a disordered region. The boxplot on the right shows the mean and median disordered content (%) in the entire DisProt repository for human proteins.

Next, we set out to interrogate the relationships between the identified cross-links and predicted protein structures from AlphaFold. To this end, we used the xiVIEW^48^ platform to compute 3D distances for 2826 intralinked residue pairs (49% of all) from 217 proteins with at least 6 intralinks excluding long loop-links; these were mapped to the respective AlphaFold models. The distribution of the distance values showed a median of 20.04 Å, which is the expected maximum distance constraint of the cross-linker^29^ but with a tail of values significantly exceeding this constraint (Figure 3A). Additionally, cross-links shorter than the median distance had higher number of cross-link spectrum matches (CSMs) compared to those exceeding the constraint (Figure 3B). As the number of CSMs can be indicative of the total abundance of the cross-links, the latter likely suggest that residues in closer proximity are cross-linked more efficiently.

To add granularity to the extent to which these models satisfy the mapped cross-links, we computed the standard deviation (SD) of the distances within each protein and ranked the models based on average distance (shorter or longer) and SD (consistent or variable) (Figure 3C, top panel). This classification revealed models that satisfy the cross-link restraint very well and others with less consistent or poor fit. Notably, overlaying the average per-residue model confidence scores (pLDDT) showed that proteins with higher discrepancy between the cross-links and the predicted structure tend to have lower AlphaFold confidence scores on average (Figure 3C, bottom panel). This confirms the utility of cross-linking data in evaluating predicted models. However, it should be also taken into consideration that low probability predictions may indicate structural flexibility related to the functional states of proteins or their presence in quaternary assemblies rather than monomers. Therefore, not all predicted monomeric models are suitable for cross-examination with cross-linking data. Additionally, several of the proteins are often mutated in this panel of cell lines in the multiplexed experiment, also contributing to the observed discrepancies due to generation of noncanonical protein products. The latter highlights the need for methods that can assess sample specific alterations in protein structures.

Disorder sequence regions are challenging for computational prediction approaches, as they are often missing in structural data used for training the models. To determine the proportion of cross-links mapping onto intrinsically disordered proteins we overlaid the cross-linked residues with disordered regions of human proteins catalogued in the DisProt repository of manually curated entries.^50^ We found that for 15% of the cross-links, at least one protein in the pair (intra or inter) was catalogued in DisProt (Figure 3D). Of these, a significant proportion of 39% represented cross-links with at least one side linked to a disordered region (Figure 3D). This likely suggests that there is no apparent positive or negative bias toward enhanced cross-linking in disordered regions considering that the average disorder content of all proteins in DisProt is 22.1% (Figure 3D, boxplot) and that each cross-link has two sides that can either reside in a disordered region.

**Figure 4:**
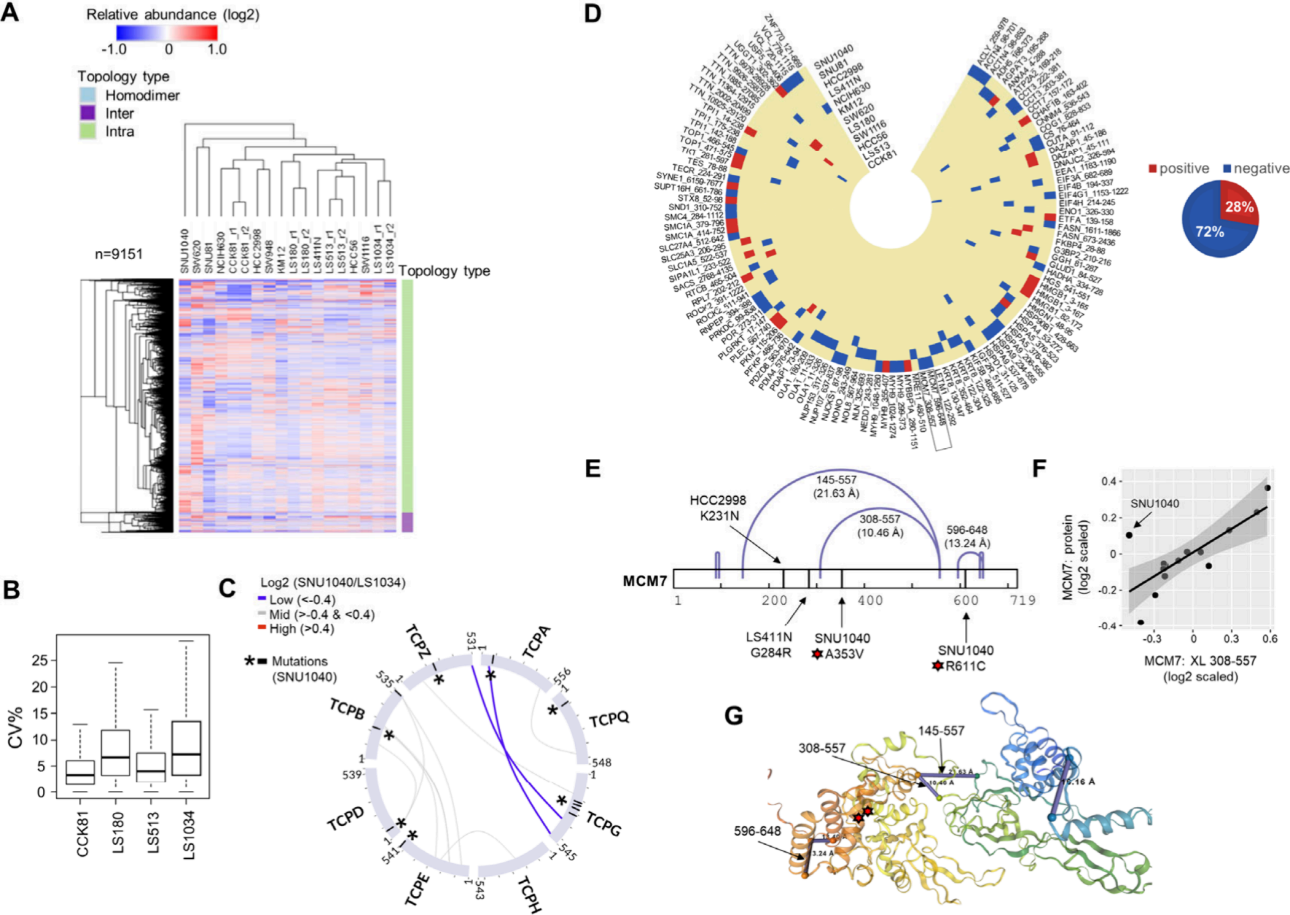
Quantitative analysis of cross-links across the colorectal cancer panel. A) Hierarchical clustering heatmap using relative abundances in log2 scale for intra- and interlinks across the different cell lines. B) The coefficients of variation (SD/mean ×100) were computed from the normalized intensities (before log2 scaling) of the aggregated residue pair data using the technical replicates of each cell line, for each residue pair. The box plots show the distribution of these CVs for each cell line. C) Circular plot of protein sequences from the chaperonin-containing T-complex with mapping of interlinks and mutations in the SNU1040 cell line. Interlinks are colored according to Log2 SNU1040/LS1034 values. D) Circular heatmap of intralinks with quantitative variations in cell lines harboring missense mutations in positions between the first and second linked residues. Quantitative values have been transformed into discrete values −1:blue and 1:red to represent negative and positive effects, respectively. E) Mapping of cross-links and mutations on MCM7 sequence. F) Scatter plot and linear regression line of log2 scaled values of the 308–557 cross-link on MCM7 (*x*-axis) versus the respective total protein abundance (*y*-axis) across cell lines; the outlier SNU1040 cell line is labeled. G) Mapping of MCM7 intralinks on the AlphaFold model. The positions of the mutations of the SNU1040 cell line are highlighted with a red star.

In addition to the qualitative characteristics of the cross-linked residues, our workflow also enabled the simultaneous comparative quantitative profiling of structural attributes across 18 samples, corresponding to 14 distinct cell lines. We quantified 9151 cross-links (including long loop-links) with no missing values (Supporting Information, Table S4) and very good reproducibility as demonstrated by the unsupervised clustering of the technical replicate samples (Figure 4A). Specifically, the median coefficient of variation was less than 15% for the vast majority of the cross-links in the cell lines that were analyzed in duplicates (Figure 4B), allowing reliable comparison of structural states.

Typically, protein interaction networks are depicted with protein nodes color-coded according to abundance differences between biological conditions.^41^ Although this is useful to highlight the abundance regulation of interacting proteins associated with a particular biological process, it does not provide resolution into potential loss or gain of interacting protein interfaces either inter- or intramolecularly. This is because the network edges are retrieved from generic repositories of known PPIs that may vary in a context specific manner. The generation of quantitative XL-MS data from our workflow enables *de novo* construction of protein networks with topological information. In this type of network, the edges, rather than the nodes, can be color-coded to identify sample specific characteristics of protein interactions. For example, we identified 11 heteromeric cross-links between 8 subunits of the chaperonin-containing T-complex (TRiC) and colored these according to the log2Ratio between a cell line with frequent mutations (SNU1040) and a cell line with no mutations (LS1034) on this complex (Figure 4C). Interestingly, we found that two of these cross-links connecting the C-terminus region of TCPG with the N- or C-terminus of TCPA and TCPZ, respectively, had lower levels in the SNU1040 cell line (Figure 4C). Notably, TCPG has four mutations in the middle of the sequence between positions 216 and 313 in the SNU1040 cell line, including a nonsense substitution at R266 (Figure 4C), which likely contribute to the partial loss of the interlinked interfaces at its C-terminus. Therefore, this type of analysis can indicate those variants with stronger impact on the integrity of protein interactions with topological detail contributing to systematic efforts in deciphering the impact of genomic variation on function.^57^

Apart from uncovering possible effects of mutations in protein–protein interactions, our quantitative cross-linking data can also be used to assess their impact on protein structures intramolecularly across different genomic backgrounds. To identify such events in a proteome-wide scale, we performed total protein abundance quantification using the linear peptides identified and used these measurements to correct the relative abundances of intralinks across cell lines by linear regression. The aim of this correction was to eliminate intracross-linking differences between cell lines that can be attributed to total protein differences and focus on the residuals of the regression that may represent net differences in structural features. For this analysis we used only missense mutations from the COSMIC^49^ database. Specifically, we focused on a subset of 1104 intralinks for which there was at least one mutation residing within the first and second linked positions in any of the cell lines. We hypothesize that these mutations may have a more significant impact on the proximity of the linked residues, therefore altering the abundance of the respective cross-link in a cell line specific manner, although we cannot rule out the possibility that mutations at any position can cause conformational changes. Next, we retrieved the regressed quantitative values only for the cell lines in which their mutations were located within the cross-link start and end positions, and additionally applied filters for regressed log2ratio >0.4 or <-0.4 and >2 × SD of the values across all cell lines. These criteria resulted in selecting 115 events of possible associations between mutations and structural variations that are summarized as a heatmap with discrete values in Figure 4D. As expected, the majority of these (72%) represented a negative association, which reflects cross-linking decrease in the presence of a mutation. For example, we found two intralinks in the MCM7 protein (308–557 and 596–648) that were within the expected distance constraint (AF model); however, they quantitatively showed lower abundances in the SNU1040 cell line which has mutations in the protein sequence between the cross-linked sites (Figure 4E). Figure 4F exemplifies the analysis performed as a scatter plot between the quantitative profile of the cross-link 308–557 versus the respective one of the total protein abundances. This demonstrated that regression analysis can help identify outliers that may result from structural alterations in particular cell lines (e.g., SNU1040). Although MCM7 is mutated in two additional cell lines at positions encompassed by the cross-link at 145–557, this did not show any considerable abundance differences, likely reflecting a negligible structural impact. Additionally, mapping the cross-links and the mutations of SNU1040 on the 3D structure of the MCM7 AF model revealed that although the two mutations are on distant positions on the protein sequence, they are found in proximity in the 3D space, which could potentially enhance their effect on the cross-link abundances (Figure 4G).

## Conclusions

The structural features of proteins determine their functions, which are often exerted through stable or transient interactions with other proteins or different biomolecules to maintain cellular processes and biological phenotypes. Integration of cross-linking mass spectrometry with other structural biology techniques and computational methods can provide insights into the spatial proximity of amino acid residues to delineate the architecture of protein assemblies. From the technical perspective, application of XL-MS in isolated proteins or small complexes has become more accessible over the years, usually producing a high number of intra- or intercross-links per protein. This is due to the reduced dynamic range of the peptides in the enriched pool of proteins, the high sensitivity of modern mass spectrometers, and the improvements in data processing algorithms for reliable identification of cross-linked peptides. However, achieving deep cross-linking coverage in whole cell samples is substantially more challenging and often requires large starting protein amounts, subcellular or protein level separation, and the use of enrichable cross-linkers. These requirements can present obstacles for large-scale cross-linking analysis that would enable comparison of the structural characteristics in different biological conditions. Multiplexing of the cross-linked peptides with isobaric tags can enable such comparisons, and as the final peptide amount is the combination of all samples, starting amounts per sample can be reduced.

Here, we have leveraged the merits of multiplexing and enrichable cross-linking to explore the qualitative and quantitative characteristics of protein interfaces in a proteome-wide scale across genomically distinct cell lines for the first time. Specifically, we combined PhoX cross-linking with TMT labeling to develop a streamline workflow (PhoXplex) for analysis of cell pellets of ∼3 million cells. In addition to the gain in sensitivity, the use of an enrichable cross-linker also significantly reduces the amount of TMT reagents required by excluding most linear peptides and making the entire process more cost-effective and feasible for large-scale proteome studies. A key component of the workflow is also the development of an open-source data analysis tool that enables TMT quantification of cross-linked peptides from the output of pLink2, a popular search engine for XL-MS data. We demonstrate the feasibility of PhoXplex in a panel of colorectal cancer cell lines where we quantified over 9000 cross-links with high reproducibility. These include a significant number of potentially novel physical interprotein associations. Mapping a large portion of the intralinks onto AlphaFold models showed the expected average length based on the linker length restraint, however with a significant degree of variation. The cross-links corroborated well with several models, but for others the agreement was poor, making them candidates for refinement. Notably, the latter largely represent models with lower prediction confidence, highlighting the value of XL-MS in model evaluation. It is possible that a number of these observed discrepancies are due to cell line specific mutations. Additionally, we found no significant over- or under-representation of cross-links onto disordered regions. Using relative cross-linking quantitation, we present a framework to study associations between genomic mutations and protein structures in a global scale exemplified by specific findings. We demonstrate the application of this approach to discover missense mutations that can lead to destabilization of protein interfaces.

Despite the very good coverage of quantified cross-links, our work also identified several limitations and areas for improvement. For example, the short length of the PhoX cross-linker seems to capture a smaller portion of interlinks compared to longer cross-linkers such as DSSO. This may also be influenced by other factors, such as the noncleavable nature of PhoX, which can affect FDR estimation and warrants further investigation. These limitations are disadvantageous for PPI mapping at large scale; however, these could be addressed by synthesis of a phospho-enrichable cross-linker with a longer and/or MS cleavable linker with minimal adjustments to the current protocol. Synthesis of heterobifunctional cross-linkers with phospho-enrichable moieties could also expand the analysis depth by targeting additional amino acids. Due to its easy implementation, here we used ultrafiltration-based peptide prefractionation to enhance MS detectability of cross-linked peptides, which surprisingly retained peptides smaller than the indicated mass. Therefore, column-based peptide size exclusion prefiltering could further improve the analysis depth by providing more efficient orthogonal enrichment of cross-links. This can be critical given that enrichable cross-linking still significantly enriches peptides with monolinks, although the number of linear peptides is drastically reduced. Moderate upscaling of the cell numbers combined with the above potential improvements could also increase detectability of low abundant cross-links. Regarding the quantitative aspects of our work, our primary aim was to evaluate the technical reproducibility of the method, particularly given the complexity and multiple stages involved, such as enrichment and cleanup, that can introduce technical variability. Therefore, incorporation of multiple biological replicates per cell line is warranted to increase the statistical robustness of the identified genome-to-structure associations in more biologically focused investigations in the future. Overall, our PhoXplex workflow paves the way for streamline analysis and deep comparative comparisons of protein interfaces at large-scale.

## Data Availability

The mass spectrometry proteomics data have been deposited to the ProteomeXchange Consortium via the PRIDE^58^ partner repository with the data set identifier PXD052480. The code used for extracting and mapping TMTpro ion intensities is available at https://github.com/DrJCWright/TMTion_Extractor.

## Supporting Information Available

Figure S1. Peptide mass distributions, cross-linked protein classification and protein–protein interaction network. A) Boxplots of peptide mass for cross-linked, monolinked and regular peptides per MS analysis run. B) Protein classification bar plots for proteins with cross-links. C) Protein–protein interaction network based on the identified interlinks found in a separate search using a concatenated shuffled decoy fasta file as false target database together with the true target database (entrapment database approach). PPIs were filtered at 10% FDR from the xiFDR output. The pie chart shows the overlap with known STRING physical interactions which are highlighted as red edges. Table S1. Cross-linked peptide pairs per MS analysis run with TMTpro un-normalized intensities. Table S2. Loop-linked peptides per MS analysis run with TMTpro un-normalized intensities. Table S3. xiFDR output for PPIs at FDR < 10%. Table S4. Unique protein residue pairs with TMTpro un-normalized, normalized and log2 scaled intensities. (ZIP)

## Abbreviations

HCD: higher-energy collisional dissociation
TMT: tandem mass tags
TBDSPP: *tert*-butyl disuccinimidyl phenyl phosphonate
XL-MS: cross-linking mass spectrometry
NHS: *N*-Hydroxysuccinimide
TCA: Trichloroacetic acid
Fe-NTA: iron-nitrilotriacetic acid
TCEP: tris-2-carboxyethyl phosphine
TEAB: triethylammonium bicarbonate
TFA: trifluoroacetic Acid
MWCO: molecular weight cutoff
DSSO: disuccinimidyl sulfoxide
PPIs: protein–protein interactions
CSMs: cross-link spectrum matches
SD: standard deviation
COREAD: colorectal adenocarcinoma
pLDDT: predicted local distance difference test
FDR: False Discovery Rate
