# Supplementary material for: PhoXplex: Combining Phospho-enrichable Cross-Linking with Isobaric Labeling for Quantitative Proteome-Wide Mapping of Protein Interfaces
Source: J Proteome Res. 2024 Oct 18;23(11):5209–20. doi: 10.1021/acs.jproteome.4c00567 (PMC11537259; doi:10.1021/acs.jproteome.4c00567)
Supplement: Supplementary file 1 — pr4c00567_si_001.zip [file pr4c00567_si_001.zip › Hoenger_et_al_supplementary_revision/Hoenger_et_al_Supporting information_revised.docx]

**Supporting information**

**PhoXplex: Combining phospho-enrichable crosslinking with isobaric labelling for quantitative proteome-wide mapping of protein interfaces**

Runa D. Hoenger Ramazanova^1,#^, Theodoros I. Roumeliotis^1,#,^*, James C. Wright^1^, Jyoti S. Choudhary^1,^*

^1^The Institute of Cancer Research, Chester Beatty Laboratories, Functional Proteomics team, London, SW3 6JB

^#^Equal contribution

*Corresponding authors

T.I.R: [theo.roumeliotis@icr.ac.uk](mailto:theo.roumeliotis@icr.ac.uk)

J.S.C: [jyoti.choudhary@icr.ac.uk](mailto:jyoti.choudhary@icr.ac.uk)

**Table of contents**

[Supplemental Figures 2](#_Toc166159575)

[Supporting tables 2](#_Toc166159576)

# **Supplemental Figures**


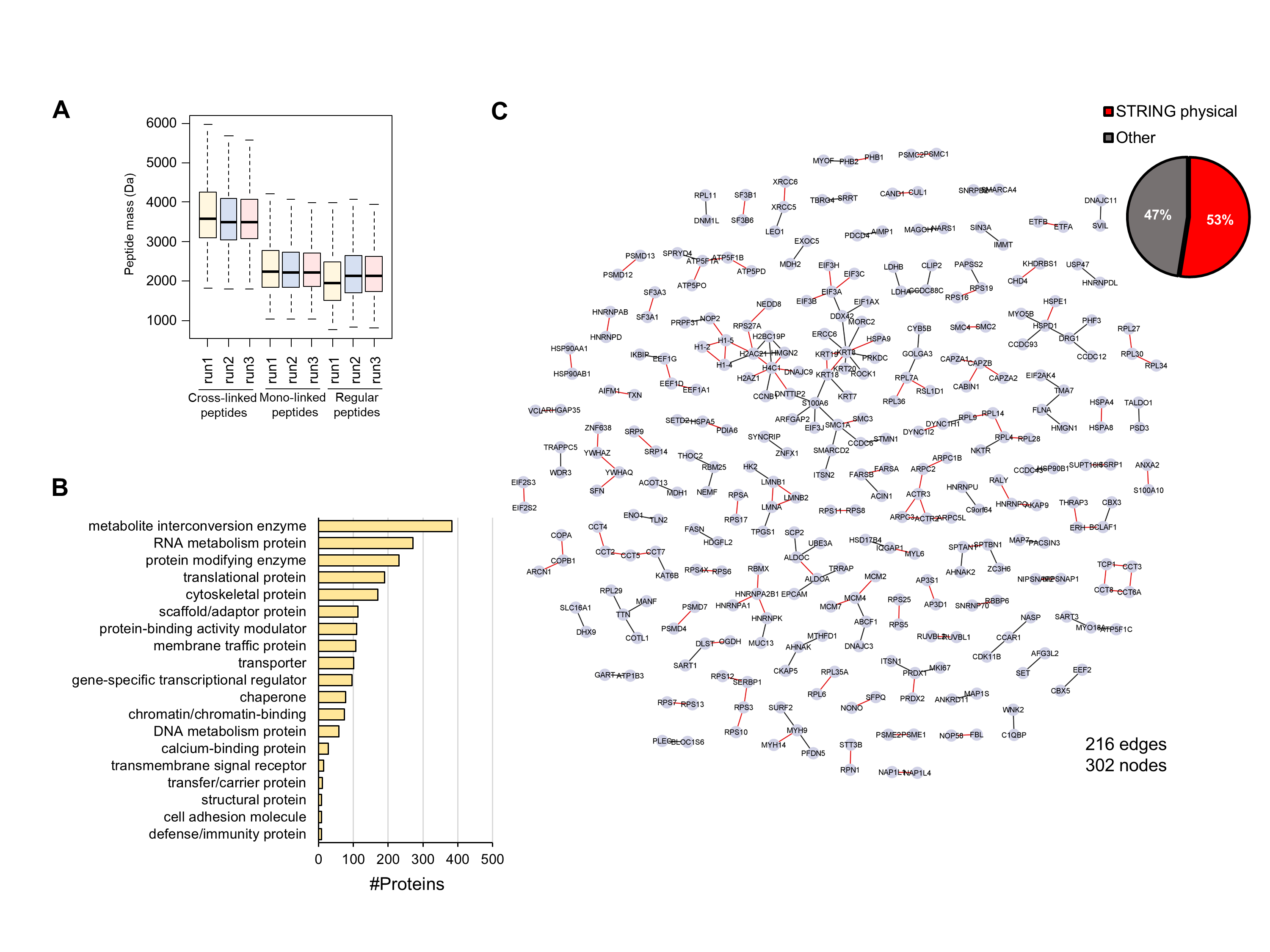


**Figure S1.** Peptide mass distributions, crosslinked protein classification and protein-protein interaction network. A) Boxplots of peptide mass for cross-linked, mono-linked and regular peptides per MS analysis run. B) Protein classification bar plots for proteins with crosslinks. C) Protein-protein interaction network based on the identified inter-links found in a separate search using a concatenated shuffled decoy fasta file as false target database together with the true target database (entrapment database approach). PPIs were filtered at 10% FDR from the xiFDR output. The pie chart shows the overlap with known STRING physical interactions which are highlighted as red edges.

# **Supporting tables**

Table S1. Cross-linked peptide pairs per MS analysis run with TMTpro un-normalized intensities.

Table S2. Loop-linked peptides per MS analysis run with TMTpro un-normalized intensities.

Table S3. xiFDR output for PPIs at FDR<10%.

Table S4. Unique protein residue pairs with TMTpro un-normalized, normalized and log2 scaled intensities.
